# Correlation of active contact location with weight gain after subthalamic nucleus deep brain stimulation: a case series

**DOI:** 10.1186/s12883-021-02383-6

**Published:** 2021-09-13

**Authors:** Katsuki Eguchi, Shinichi Shirai, Masaaki Matsushima, Takahiro Kano, Kazuyoshi Yamazaki, Shuji Hamauchi, Toru Sasamori, Toshitaka Seki, Kenji Hirata, Mayumi Kitagawa, Mika Otsuki, Tohru Shiga, Kiyohiro Houkin, Hidenao Sasaki, Ichiro Yabe

**Affiliations:** 1 Department of Neurology, Faculty of Medicine, Graduate School of Medicine, Hokkaido University, Kita 15, Nishi 7, Kita-ku, 060-8638 Sapporo, Japan; 2 Department of Neurosurgery, Faculty of Medicine, Graduate School of Medicine, Hokkaido University, Kita 15, Nishi 7, Kita-ku, 060-8638 Sapporo, Japan; 3 Department of Neurosurgery, Sapporo Azabu Neurosurgical Hospital, Kita 22, Higashi 1, Higashi-ku, 065-0022 Sapporo, Japan; 4 Department of Diagnostic Imaging, Faculty of Medicine, Graduate School of Medicine, Hokkaido University, Kita 15, Nishi 7, Kita-ku, 060-8638 Sapporo, Japan; 5 Sapporo Teishinkai Hospital, Kita 33, Higashi 1, Higashi-ku, 065-0033 Sapporo, Japan; 6 Faculty of Health Sciences, Graduate School of Health Sciences, Hokkaido University, Kita 15, Nishi 7, Kita-ku, 060-8638 Sapporo, Japan; 7 Department of Nuclear Medicine, Faculty of Medicine, Graduate School of Medicine, Hokkaido University, Kita 15, Nishi 7, Kita-ku, 060-8638 Sapporo, Japan

**Keywords:** Deep brain stimulation, Parkinson’s disease, Subthalamic nucleus, Weight gain

## Abstract

**Background:**

Weight gain (WG) is a frequently reported side effect of subthalamic deep brain stimulation; however, the underlying mechanisms remain unclear. The active contact locations influence the clinical outcomes of subthalamic deep brain stimulation, but it is unclear whether WG is directly associated with the active contact locations. We aimed to determine whether WG is associated with the subthalamic deep brain stimulation active contact locations.

**Methods:**

We enrolled 14 patients with Parkinson’s disease who underwent bilateral subthalamic deep brain stimulation between 2013 and 2019. Bodyweight and body mass index were measured before and one year following the surgery. The Lead-DBS Matlab toolbox was used to determine the active contact locations based on magnetic resonance imaging and computed tomography. We also created sweet spot maps for WG using voxel-wise statistics, based on volume of tissue activation and the WG of each patient. Fluorodeoxyglucose-positron emission tomography data were also acquired before and one year following surgery, and statistical parametric mapping was used to evaluate changes in brain metabolism. We examined which brain regions’ metabolism fluctuation significantly correlated with increased body mass index scores and positron emission tomography data.

**Results:**

One year after surgery, the body mass index increase was 2.03 kg/m^2^. The sweet spots for WG were bilateral, mainly located dorsally outside of the subthalamic nucleus (STN). Furthermore, WG was correlated with increased metabolism in the left limbic and associative regions, including the middle temporal gyrus, inferior frontal gyrus, and orbital gyrus.

**Conclusions:**

Although the mechanisms underlying WG following subthalamic deep brain stimulation are possibly multifactorial, our findings suggest that dorsal stimulation outside of STN may lead to WG. The metabolic changes in limbic and associative cortical regions after STN-DBS may also be one of the mechanisms underlying WG. Further studies are warranted to confirm whether dorsal stimulation outside of STN changes the activities of these cortical regions.

## Background

Subthalamic nucleus deep brain stimulation (STN-DBS) is an established and effective treatment strategy for advanced Parkinson’s disease (PD) [1]. However, STN-DBS is associated with several adverse effects, of which, weight gain (WG) is one of the most common, although the underlying mechanisms have not been fully elucidated. Previous reports have suggested that WG is associated with lowered resting energy needs [2, 3], fewer motor complications (especially dyskinesia) [4–6], changes in eating behaviors [7, 8], and hormonal factors [9, 10]. However, these factors do not completely explain WG, which suggests that WG following STN-DBS is a multifactorial process [11].

The locations of electrodes affect the clinical outcomes and adverse effects of STN-DBS [12, 13]. Based on animal studies [14, 15] and studies examining the human brain using diffusion tensor imaging [16–18], the STN is divided into three functional subregions: the sensorimotor, associative, and limbic regions. Previous studies have suggested that superior motor improvement is achieved by stimulating the sensorimotor area, which is located in the dorsolateral part of the STN and is linked to the primary motor and supplementary motor cortices [17, 19–21]. Nevertheless, an increased risk of neuropsychiatric side effects may be associated with stimulation of the limbic area, which is present in the anteromedial part of the STN [17, 22]. However, few studies have evaluated whether WG is directly correlated with the stimulation location during STN-DBS. Therefore, this study prospectively evaluated patients who underwent STN-DBS for PD and aimed to determine whether WG was correlated with the positions of the active contacts and distribution of volume of tissue activation (VTA).

In addition, several previous studies using fluorodeoxyglucose-positron emission tomography (FDG-PET) have shown a correlation between behavioral or cognitive changes and metabolic changes in specific brain regions following STN-DBS [23, 24]. We hypothesized that regions in which metabolic changes are seen, could help identify the regions in the STN correlated with WG after STN-DBS. Therefore, we also evaluated whether WG was associated with altered glucose metabolism in specific brain regions.

## Methods

### Patients

This prospective study recruited patients with PD, who underwent bilateral STN-DBS surgery, at Hokkaido University Hospital between 2013 and 2019. PD diagnoses were based on the UK Parkinson’s Disease Society Brain Bank criteria [25]. We recruited 16 patients with PD who underwent bilateral STN-DBS surgery; we then excluded one patient who withdrew from participating and another who required electrode removal due to an infection. Thus, this study analyzed data from 14 patients, including 12 women. The median age at DBS surgery was 62.5 (55.5–68) years, and the median disease duration at DBS surgery was 14.3 (12–20.5) years. The eligibility for DBS surgery was determined based on the guidelines of the International Parkinson and Movement Disorders Society [26]. All patients received bilaterally implanted quadripolar (from contact number ‘0’ for the most ventral contact to ‘3’ for the most dorsal one) DBS electrodes (3387; Medtronic, Minneapolis, MN, USA).

This study was conducted following the 1964 Declaration of Helsinki and its later amendments and was approved by the ethics panel of the institutional review board of Hokkaido University Hospital. All patients provided written informed consent before their inclusion in the study.

### Clinical assessment

Clinical assessments were conducted at the preoperative baseline and one year following surgery. Bodyweight and height were measured and used to calculate the body mass index (BMI, kg/m^2^). Motor symptoms were assessed using the Unified Parkinson’s Disease Rating Scale (UPDRS) part III in the MedOff state at baseline and in the MedOff and DBS-on state at one year following surgery. Dyskinesia was also evaluated using items 32 and 33 of the UPDRS part IV. Neuropsychological evaluations were based on the Mini Mental State Examination (MMSE), Frontal Assessment Battery (FAB), Apathy Scale [27], and Patient Health Questionnaire-9 [28]. The levodopa-equivalent daily dose (LEDD) [29] was calculated at both baseline and one year following DBS surgery. Brain metabolism was evaluated using FDG-PET at baseline and one year following surgery. The DBS stimulation parameters were recorded at one year following surgery and included the stimulation voltage, pulse width, frequency, and active contact location.

### Assessing the active contact positions

The active contact positions were evaluated using Lead-DBS, which is a validated Matlab toolbox [30, 31] that was implemented using Matlab 2019b (MathWorks, Natick, MA, USA). Preoperative results were obtained via T2-weighted magnetic resonance imaging (MRI, slice thickness: 1 mm; echo time: 222 ms; repetition time: 2,000 ms), and postoperative results were obtained via computed tomography (CT). The preoperative MRI and postoperative CT images were co-registered using advanced normalization tools [32] and then nonlinearly normalized into Montreal Neurological Institute (MNI) standard space (MNI_ICBM_2009b_NLIN_Asym). The DBS electrodes were automatically reconstructed using the TRAC/CORE algorithm [31] and manually refined to evaluate their coordinates in MNI space. If more than one contact was used for the stimulation, the mean coordinates of all active contacts were recorded. The positional relationships between the active contacts and the STN were assessed using the DISTAL atlas [33], which is a composite atlas based on histology, structural connectivity, and manual segmentations of a multimodal brain template normalized in MNI space.

### Calculation of volume of tissue activation

VTA calculations were performed using a finite element method approach [34], implemented in Lead-DBS based on the stimulation settings of each patient. The spread of the electric field was estimated for homogenous tissue with a conductivity of 0.14 S/m. The threshold of VTA was at the electric field isolevel of 0.2 V/mm.

### Creation of sweet spot map for WG

In this study, we assessed the sweet spot for WG after DBS using Lead-Group [35]. Each voxel of each patient’s VTA was assigned an increase in BMI. Subsequently, all VTAs were pooled across patients and the mean increase in BMI was obtained for each voxel. To identify voxels with a significantly higher increase in BMI than the average increase of BMI of all VTAs that did not stimulate that particular voxel, a two-sample t-test was performed. This test yielded a t-statistic for every voxel that was then displayed as 3D statistical maps, which we referred to as sweet spot maps. Significant voxels (uncorrected, p < 0.05) were visualized on sweet spot maps for WG. Only those voxels that were at least covered by n = 3 VTAs were considered in this analysis.

### FDG-PET acquisition and preprocessing

PET data were obtained using either a GEMINI TF64 (Philips, Amsterdam, Netherlands) PET-CT scanner or a Biograph 64 (Siemens, Munich, Germany) TruePoint PET-CT scanner for different patients. Time-of-flight technology was used with GEMINI TF64 but not with Biograph 64. The manufacturer of the DBS system warns that stimulation intensity may increase during CT and recommends turning off stimulation during the scan. In our institution, turning off DBS stimulation is required during scanning with a PET-CT scanner to avoid an increase in stimulation intensity and incurring any side effects. Therefore, in this study, a PET was performed in the MedOn at baseline and in the MedOn and DBS-off state post-operation. DBS stimulation was turned off 30–60 min before PET. Patients were instructed to fast overnight before PET [18]. F-FDG (4.5 MBq/kg) was administered intravenously, and serum glucose was measured to exclude the patients showing fasting hyperglycemia (> 150 mg/dL). The images were acquired 60 min following FDG administration for 10-min emission scanning. Images were reconstructed using ordered subset expectation–maximization. The PET images were preprocessed using statistical parametric mapping (SPM12; Wellcome Department of Cognitive Neurology, London, UK) on Matlab 2019b. The PET images were initially subjected to affine and nonlinear spatial normalization into the MNI brain space, although we used an FDG-PET-specific template that was described by Della Rosa et al. [36] instead of the default H_2_O-SPM template [15]. The normalized images were then smoothed using an 8-mm isotropic Gaussian filter to compensate for individual anatomical variability. Finally, we created a percent signal change map (PSC map) using the following formula:

$$\mathrm{PSC}=\left({\mathrm V}_{1\mathrm y}-{\mathrm V}_{\mathrm b}\right)\;\times\;100/{\mathrm V}_{\mathrm b}$$

where, V_1y_ and V_b_ represent voxel values at one year following surgery and baseline, respectively.

### Statistical analysis

Scores are reported as the median ± interquartile range (IQR), and nonparametric analyses were used based on the small sample size. Preoperative and postoperative values for BMI, UPDRS part III and IV scores, neuropsychological data, and LEDD values were compared using the Wilcoxon signed-rank test, and p < 0.001 was considered as statistically significant using Bonferroni correction. Stimulation voltage and active contact coordinates for the left and right sides were compared using Wilcoxon’s rank-sum test. A multivariate regression analysis was performed with WG as the dependent variable and contacts’ coordinates that showed a correlation with WG, age, body weight before surgery, sex, stimulation voltage, frequency, pulse width, dyskinesia reduction (scores of UPDRS part IV items 32 + 33), and LEDD reduction as independent variables to adjust for any possible confounders (p < 0.05 was considered as statistically significant). Statistical analyses were carried out using JMP Pro software (version 14; SAS Inc., Cary, North Carolina, USA).

The PET data were analyzed using SPM12 (Wellcome Department of Cognitive Neurology, London, UK). To identify which brain regions’ metabolism change correlated significantly with increased BMI scores, a general linear model was tested at each voxel with the BMI score as a covariate using a PSC map. LEDD was included in the SPM analysis as a covariate. A voxel-level threshold of *P* < 0.05 (family-wise error corrected for multiple comparisons) was used to assess the SPM *t* values. If statistical significance was not reached, we performed the same analysis with a voxel-level threshold of *P* < 0.005 (uncorrected for multiple comparisons), considering the small sample size and the study’s exploratory nature. Only clusters containing > 100 voxels were reported.

## Results

### Clinical outcomes

Table 1 shows the clinical values at preoperative baseline and one year following DBS surgery. A significant motor improvement was observed one year post-surgery, based on the decreased UPDRS part III scores in the MedOff state. A non-significant trend toward decreased values for items 32 and 33 of the UPDRS part IV was also observed. Bodyweight and BMI values increased significantly following STN-DBS. The neuropsychological assessments revealed a small but statistically significant increase in the FAB score but no significant changes in the other scales. There was a non-significant trend of decrease in LEDD after DBS surgery.

**Table 1:** Clinical values at baseline and 1-year follow-up

	Baseline	1-year Follow-up	*p*-value
Bodyweight	55.8 (49.7–63.6)	60.7 (53.1–69.6)	< 0.001
BMI	23.1 (20.1–25.7)	24.5 (22.0–29.1)	< 0.001
UPDRS part III (MedOn)	14 (10–18)	8 (4.5–19.3)	0.13
UPDRS part III (MedOff)	40 (33.8–44.3)	16 (11.8–23)	< 0.001
UPDRS part IV (items 32 + 33)	1.5 (0–4)	1 (0–2.25)	0.12
MMSE	29 (27.8–30)	29 (28–30)	0.3
FAB	14.5 (14.5–17.5)	15.5 (13.8–17.3)	0.023
Apathy scale	13.5 (7.5–16)	14 (4.8–16.3)	0.47
PHQ-9	5 (4–8)	4 (0–7.8)	0.43
LEDD	689 (363–748.5)	418.5 (297.3–656.3)	0.085

Scores are reported as the median (interquartile range)

*BMI* body mass index; *FAB* Frontal Assessment Battery; *LEDD* levodopa-equivalent daily dose; *MedOff* a condition without intaking medication; *MedOn* a condition requiring intaking medication. *MMSE* Mini Mental State Examination; *PHQ-9* Patient Health Questionnaire-9; *UPDRS* Unified Parkinson’s Disease Rating Scale

### Stimulation parameters and active contact positions

**Fig. 1:**
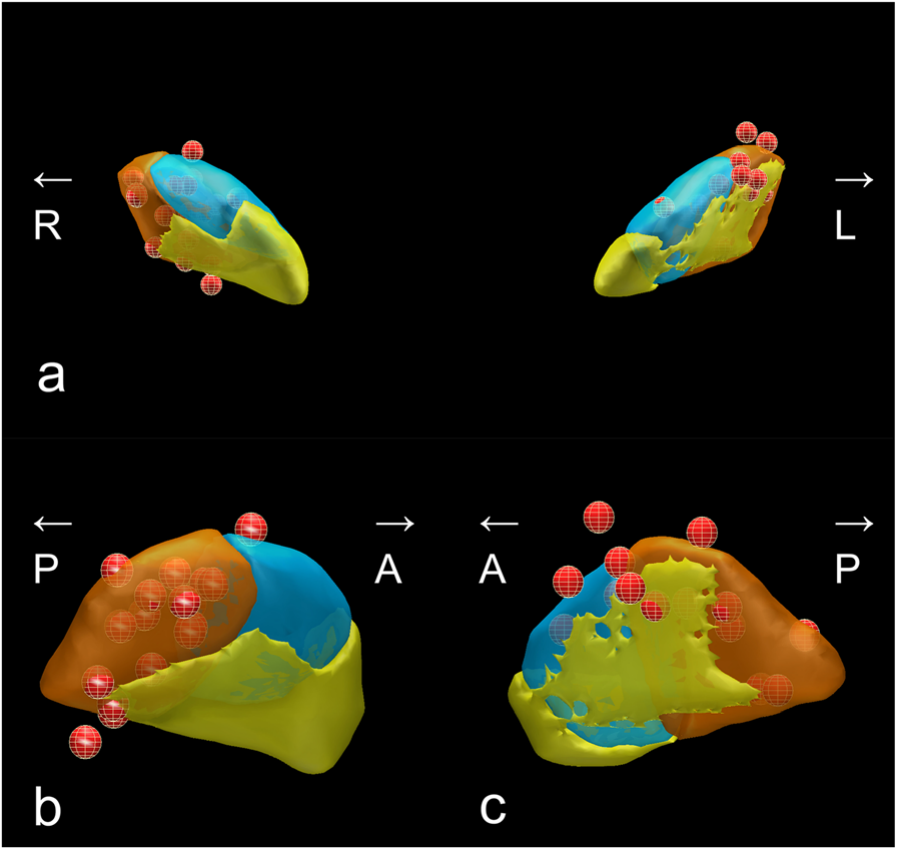
Meshed red points represent positions of active contacts. (**a**) Anterior view of the bilateral lead locations for all 14 patients as well as the subthalamic nucleus (STN), as defined using the DISTAL atlas in Montreal Neurological Institute space [33]. The functional subregions of the STN are highlighted (sensorimotor STN in copper, associative STN in blue, and limbic STN in yellow). The lateral views are shown for the right STN (**b**) and the left STN (**c**). This figure was created using Lead Group [35]. R, right; L, left; A, anterior; P, posterior

Each patient’s stimulation parameters at one year following DBS surgery are shown in Table 2. The stimulation intensities were similar between the right and left sides. Figure 1 shows the electrode locations for the STN of all patients, as defined using the DISTAL atlas and MNI space. The median coordinates of the right-side active contacts were 12.5 (11.7–13.5) mm on the X-axis, -14.5 (-13.5 to -14.9) mm on the Y-axis, and − 6.4 (-5.6 to -7.6) mm on the Z-axis. The mean left-side coordinates were − 13.6 (-11.5 to -14.2) mm on the X-axis, -13.6 (-11.8 to -15.6) mm on the Y-axis, and − 6.0 (-5.1 to -6.8) mm on the Z-axis. The X-, Y-, and Z-axis coordinates were not significantly different between the right and left sides, although the left-side Y-axis coordinates had a larger IQR than the right-side Y-axis coordinates.

**Table 2:** BMI change from baseline and stimulation parameters at one year following surgery

	Disease duration at DBS surgery (year)	Change of BMI from baseline	Active contacts		Frequency (Hz)		Stimulation Intensity (V)		Pulse width (µs)	
Subject No.			Right	Left	Right	Left	Right	Left	Right	Left
1	8	2.3	C(+)1(-)2(-)	C(+)2(-)	130	130	1.5	2.2	60	60
2	18	3.8	C(+)1(-)	C(+)2(-)	130	130	1.2	1.2	60	60
3	23	3.2	C(+)1(-)2(-)	C(+)2(-)	130	130	2.6	1.8	60	60
4	8	1.0	C(+)3(-)	C(+)3(-)	130	130	1	1	60	60
5	9	-0.2	C(+)1(-)	C(+)1(-)	130	130	1	2	60	60
6	13	-0.2	C(+)2(-)	0(+)1(-)	130	130	2.8	3.2	60	150
7	13	4.6	C(+)2(-)	C(+)2(-)	60	60	2.2	3.7	60	90
8	14	1.3	1(+)2(-)	C(+)2(-)	130	130	3.4	3.2	150	90
9	13	2.8	C(+)2(-)	C(+)3(-)	130	130	3	3	60	60
10	15	3.7	C(+)2(-)	C(+)2(-)	130	130	2.8	2.8	90	90
11	6	0.7	C(+)2(-)	C(+)2(-)	130	130	1.4	1.4	90	90
12	11	2.4	C(+)2(-)	C(+)2(-)	130	130	2.1	1.7	60	60
13	8	2.3	C(+)1(-)	C(+)2(-)	130	130	1.8	3	60	60
14	8	0.7	C(+)2(-)	C(+)1(-)	130	130	1.9	2	60	60

### Sweet spot map for WG

**Fig. 2:**
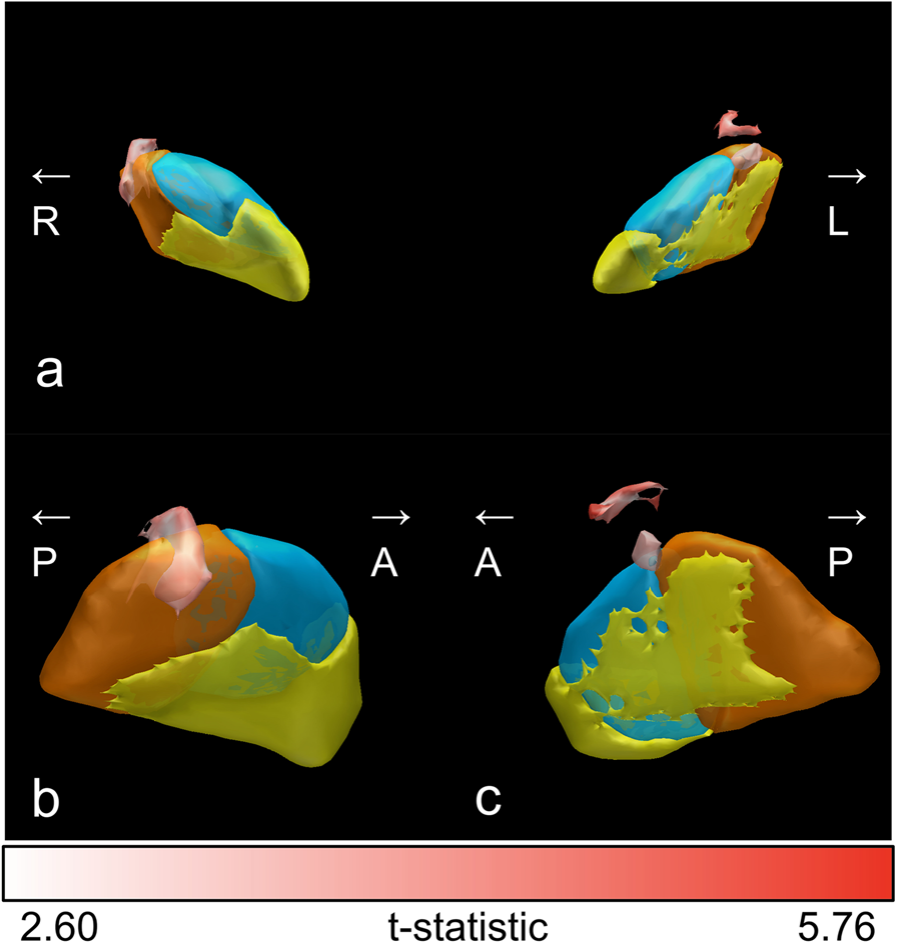
The sweet spot map for weight gain based on the results of the voxel-wise statistical analysis. (**a**), (**b**), and (**c**) show the anterior view of the bilateral section of the STN, lateral view of right STN, and lateral view of left STN, respectively. The functional subregions of the STN are highlighted (sensorimotor STN in copper, associative STN in blue, and limbic STN in yellow). The color bar shows t-statistic of the sweet spot map. This figure was created using Lead Group [35]. R, right; L, left; A, anterior; P, posterior

Figure 2 shows the sweet spot map for the WG results of voxel-wise statistical analysis using VTA. The sweet spots for WG were mainly located dorsally and outside of the STN, bilaterally.

### Correlations with increased BMI

We could not obtain a statistically significant model on multivariate regression analysis (P = 0.11).

### PET image analysis

**Fig. 3:**
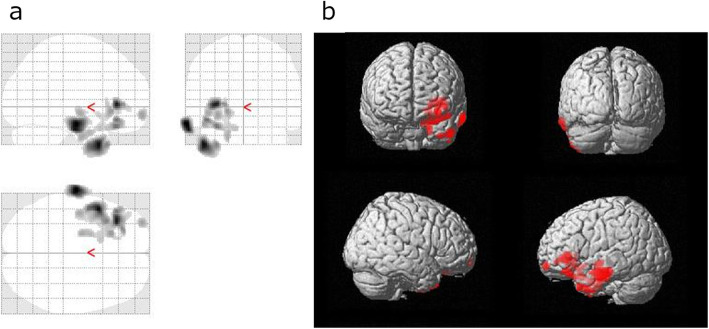
Positive correlations between weight gain and brain metabolism detected using fluorodeoxyglucose-positron emission tomography were based on three orthogonal views (**a**) and a three-dimensional brain surface projection (**b**)

**Fig. 4:**
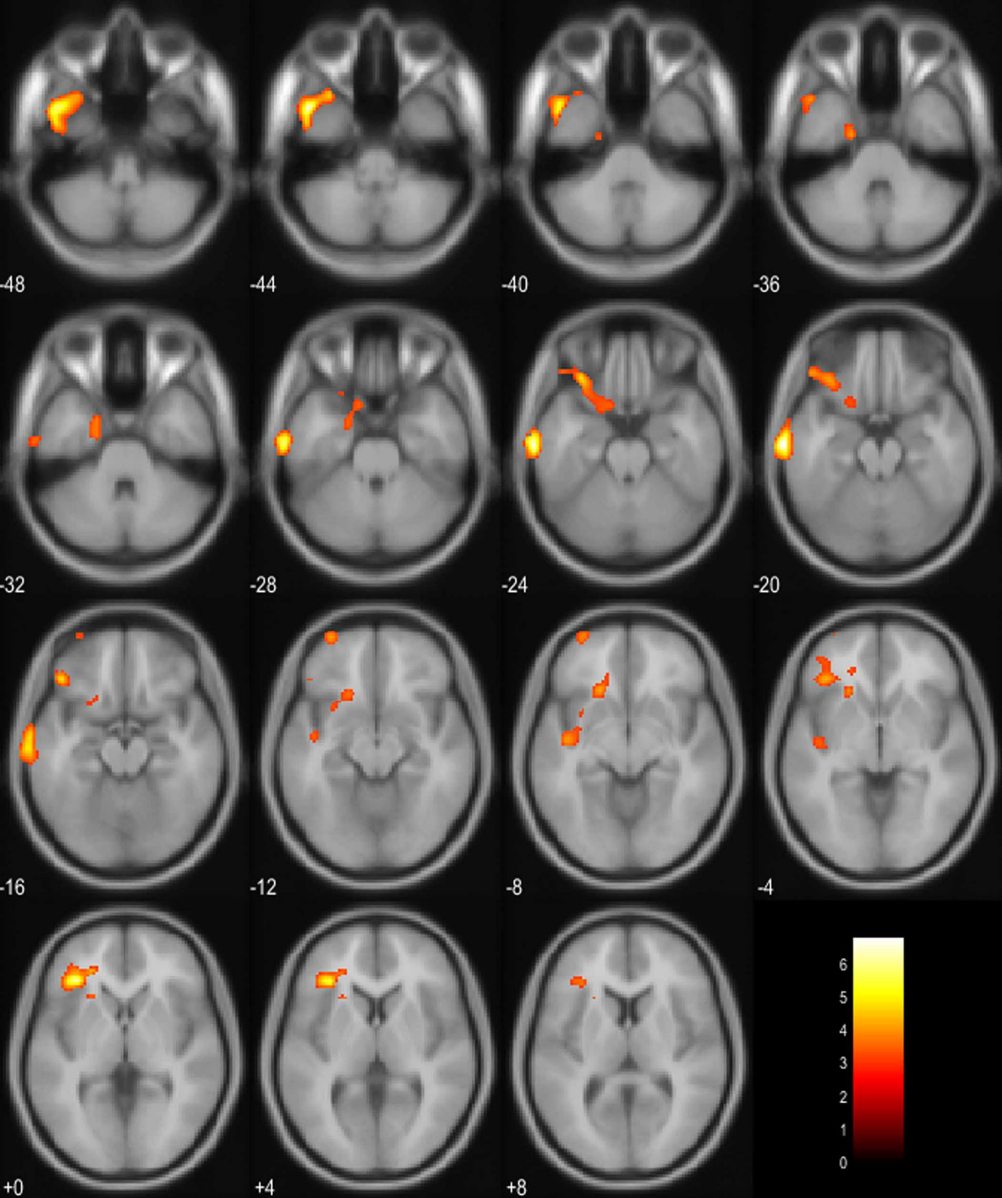
Projection of voxels that show positive correlations between weight gain and increased brain metabolism on axial template brain slices. The yellow marked regions indicate areas with significantly positive correlation between weight gain and brain metabolism

We analyzed PET data from 13 patients since one patient’s PET data were missing. A GEMINI TF64 PET-CT scanner was used for 12 patients, while a Biograph 64 TruePoint PET-CT scanner was used for the remaining patient. None of the voxels were significant at a voxel-level threshold of *P* < 0.05 (family-wise error corrected for multiple comparisons). However, at a threshold of *P* < 0.005 (uncorrected for multiple comparisons), we identified several clusters with positive correlations between WG and increased metabolism in the left hemisphere (Table 3; Figs. 3 and 4). The correlations were observed in the left middle temporal gyrus, inferior frontal gyrus, lateral orbital gyrus, anterior orbital gyrus, and planum polare. We did not observe any negative correlations between WG and brain metabolism in these various areas.

**Table 3:** Correlations between weight gain and increased brain metabolism at various locations

	Coordinates				
Region	X	Y	Z	Peak T value	No. of Voxels
Left middle temporal gyrus	−68	−16	−20	6.8	575
Left triangular part of the inferior frontal gyrus	−34	32	2	6.26	401
Left lateral orbital gyrus	−44	32	−16	4.83	734
Left anterior orbital gyrus	−32	62	−10	4.11	103
Left planum polare	−42	-12	−8	4	107

The uncorrected thresholds were significant (*P* < 0.005) at the voxel level. Only clusters with > 100 voxels are reported

## Discussion

This study showed a mean BMI increase of 2 kg/m^2^ at 1 year following DBS surgery; this concurs with previous findings [37]. Furthermore, the mean active contact coordinates in our study were similar to the preferred coordinates for motor improvement in previous studies [38]. The sweet spot for WG was located in the dorsal part of the STN and dorsally outside the STN on both sides. We could not detect a significant correlation between WG and dyskinesia reduction; previous findings using multivariate regression analysis remain conflicting [4–6, 38].

In this study, sweet spots for WG were located in both, the dorsal part of the STN and dorsal to the STN on both sides. These findings agree with those of a previous report, which indicated that active contacts located in the zona incerta (dorsal to the STN) were correlated with increased appetite after STN-DBS [39]. The zona incerta contains neurons expressing melanin-concentrating hormone, which is involved in the regulation of feeding [40]. Thus, our finding of the location of the sweet spot for WG may be explained by the stimulation of the zona incerta and neurons that express melanin-concentrating hormone.

We observed correlations between WG and increased metabolism in several brain regions that belonged to the limbic and associative areas. This finding may explain another mechanism underlying WG following STN-DBS. The STN plays an important role in reward processing [41], and several studies have indicated that the STN is involved in controlling appetite and eating behaviors. For instance, a study on non-human primates illustrated that STN activity increased during food reward anticipation and delivery [42]. In humans, stroke or tumors affecting the STN causes hyperphagia and increases appetite [43, 44], and abnormal eating behaviors have been reported following STN-DBS [7, 45–49]. These findings from previous studies suggest that behavioral changes after STN-DBS may cause WG. A previous FDG-PET study showed that WG after STN-DBS was correlated with increased metabolism in the limbic and associative regions, including the orbitofrontal cortex, lateral and medial parts of the temporal lobe, anterior cingulate cortex, and retrosplenial cortex [38]. Other PET and functional MRI studies have also suggested that a broad network of limbic and paralimbic structures mediate the desire for food [50–57]. This network is thought to integrate sensory information with the cognitive desire for food, and induces behaviors that aim to obtain food [58, 59]. Regions with increased brain metabolism in our study were also associated with the processing of desire for food; changes of the activities in the limbic and associative areas may modify food-related behavior, ultimately causing WG. However, our sweet spot map for WG in this study did not contain the limbic STN, which has strong structural connectivity with the limbic brain region. The hyperdirect pathways, connecting the cortex and the STN, pass from the motor and associative cortex through the dorsal area of STN; however, those from the limbic cortex pass through the anterior area of STN [33]. The results of the sweet spot analysis were inconsistent with the FDG-PET results. However, a previous study on non-human primates, using anterograde tracers, revealed overlapping projections from M1 to the dorsolateral STN and from the prefrontal cortical areas to the anterior, ventral, and medial half of the STN [15]. Another study using diffusion tensor imaging found overlaps between the subregions within the STN, and their gradual transition into each other [18]. These findings suggest that neurons in each STN subregion receive multiple inputs and integrate information from different cortical regions. Stimulation to hyperdirect pathways passing the dorsal area of the STN may influence the integration of information from different cortical regions, and alter the activities of limbic cortex. A larger prospective study with correction for multiple comparisons is warranted to confirm this hypothesis.

We observed that WG was correlated with increased brain metabolism only on the left side. Several studies have also indicated that unilateral STN-DBS causes WG [60, 61], although the laterality of this relationship remains unclear. Further studies are warranted to determine whether right-side STN-DBS may influence metabolism in limbic and associative regions, and subsequently cause WG.

In this study, LEDD did not decrease significantly following STN-DBS. This may be linked to the fact that some patients were tremor dominant or experienced severe side effects with dopaminergic therapy. These patients were treated with a relatively low dose of dopaminergic medication and did not need a reduction in dopaminergic medication following DBS surgery. Indeed, the median LEDD value in this study was lower than that reported in previous studies. In addition, an aggressive reduction in dopaminergic medication following DBS may incur behavioral side effects, such as apathy and depression [62]. We reduced the medication dose carefully to prevent these behavioral side effects; this may be another reason why LEDD did not show any significant reduction following DBS surgery. Although a previous study reported significant correlation between WG and LEDD reduction [63], we could not find any correlation after multivariate regression analysis in this study. This may be attributed to the relatively small magnitude of LEDD reduction. However, future studies with larger sample sizes are needed to evaluate the effect of LEDD reduction on WG after STN-DBS.

This study has several limitations. First, we did not assess eating habits or daily food intake. Thus, future studies must confirm whether WG is caused by stimulation of the limbic area, which induces changes in eating behaviors, using preoperative and postoperative data on eating behaviors and food intake. Second, we did not assess hormonal factors or swallowing function, which could have confounded our analyses. Third, we obtained PET data only in an “off DBS” state after the surgery, owing to safety reasons. Therefore, it is difficult to attribute the changes in brain metabolism after DBS to the plasticity of the neural circuit or the washout process of therapeutic DBS. Fourth, we were not able to obtain a statistically significant model on multivariate regression analysis. This may be related to a relatively smaller number of patients compared to the number of independent variables. Further studies with a larger number of patients are required to confirm the correlation between active contact location and WG.

## Conclusions

Although the mechanisms underlying WG following subthalamic deep brain stimulation are possibly multifactorial, our findings suggest that stimulation to the dorsal outside of the STN may lead to WG. Additionally, the metabolic changes in limbic and associative cortical regions following STN-DBS also correlated with WG. Stimulation to hyperdirect pathways passing through the dorsal area of the STN may influence the integration of information from different cortical regions and change the functions of the limbic and associative cortex. Further investigations are warranted to confirm this hypothesis by accurately assessing eating behaviors and food intake.

## Data Availability

The dataset(s) used and/or analyzed during the current study are available from the corresponding author on reasonable request.

## Abbreviations

BMI: Body mass index
CT: Computed tomography
FAB: Frontal Assessment Battery
LEDD: Levodopa-equivalent daily dose
MMSE: Mini Mental State Examination
MNI: Montreal Neurological Institute
MRI: Magnetic resonance imaging
PD: Parkinson’s disease
PHQ-9: Patient Health Questionnaire-9
PSC: Percent signal change
UPDRS: Unified Parkinson’s Disease Rating Scale
VTA: Volume of tissue activation
WG: Weight gain
